# Bacterial Symbionts May Prove a Double-Edged Sword for the Sharpshooter

**DOI:** 10.1371/journal.pbio.0040218

**Published:** 2006-06-06

**Authors:** Liza Gross

Though scientists recently downsized estimates of insect species from over 30 million to around 4 million to 6 million, it's safe to say that insects are among Earth's most numerous and diverse group of organisms. At least some of this success derives from mutually beneficial interactions with bacteria, established as long as 270 million years ago, that allow insects to thrive in otherwise unsuitable niches. These symbiotic bacteria—called *endo*symbionts because they live inside cells—synthesize essential nutrients for their hosts, while the insects provide an ecological niche for the bacteria.

In a new study, Dongying Wu, Jonathan Eisen, Nancy Moran, and colleagues used comparative genome analysis to investigate the inner life of a widespread agricultural pest, the glassy-winged sharpshooter *(Homalodisca coagulata)*. As the vector of Pierce disease (caused by the *Xylella fastidiosa* bacterium), the sharpshooter poses a major threat to California crops, having already destroyed an estimated $14 million of Southern California grapevines. It lives on xylem sap—which distributes salts and water throughout the plant but provides very few organic nutrients to the insect—and houses two endosymbionts, *Baumannia cicadellinicola* and *Sulcia muelleri*. These two unrelated endosymbionts, they show, play distinct, complementary roles that supplement their host's diet.

Endosymbionts were first described through microscopy in the early 20th century, but their metabolic secrets have only recently come to light by making inferences about gene function based on genome analysis. After sequencing the *B. cicadellinicola* genome, Wu et al. built a genome-based evolutionary tree (called a phylogenetic tree) for *B. cicadellinicola* and related endosymbiont species. *B. cicadellinicola* was grouped together with endosymbionts of aphids *(Buchnera)*, tsetse flies *(Wigglesworthia)*, and ants *(Blochmania)*, in keeping with other genomic studies, but was “the deepest branching symbiont,” suggesting it had separated from the group earlier.

The endosymbiont genomes shared a number of features, including reduced genome size, fewer guanine-cytosine (G-C) than adenine-thymine (A-T) base pairs, and rapidly evolving proteins. These trends can be found in endosymbionts arising across all the major bacterial groups, challenging biologists to figure out the mechanisms driving them. One explanation centers on changes that arise by chance in small populations (called drift); the other centers on a higher rate of mutations stemming from the loss of DNA repair systems. Interestingly, though some researchers favor one hypothesis over the other, analysis of the *B. cicadellinicola* genome suggests both are important, but act at different levels. The differences between endosymbionts and free-living species appear to be due to genetic drift. Differences among symbionts, however, appear to be due to differential loss of DNA repair genes.

The researchers argue that the nature of some of *B. cicadellinicola*'s other genome features (such as G-C content) and position on the evolutionary tree can help fill in missing gaps between free-living and intracellular species. And because its proteins are evolving more slowly than those of other endosymbionts—making it more likely that similar sequences really do reflect evolutionary relatedness rather than artifacts that occur when only fast-evolving sequences are used—inferences about the evolutionary events promoting intracelluarity can be made with more confidence.

As for *B. cicadellinicola*'s metabolic capabilities, the researchers found a “relatively limited repertoire.” It lacks the necessary genes to support sugar metabolism, and probably does not use sugar as energy source since almost none is present in the xylem sap diet. Energy likely comes from using the more abundant amino acids, one of the main types of organic compounds present in xylem. In return, it provides the insect with a range of vitamins and cofactors (required for enzyme activity). But surprisingly, *B. cicadellinicola* lacks key enzymes needed to synthesize essential amino acids. Obviously, the insect must get the missing essential nutrients from somewhere, and the most likely candidate, they reasoned, was the other bacterium known to reside in its cells, *S. muelleri*.

They went back to the sequences that didn't assemble with *B. cicadellinicola*, realizing they might contain part of the *S. muelleri* genome, and found genes required for the synthesis of several essential amino acids. Though some of the sequences belonged to other bacterial species, the vast majority required for amino acid synthesis belonged to *S. muelleri*. Since the sample of this bacterium's genome contained very few genes related to vitamin or cofactor synthesis, Wu et al. concluded that the two endosymbionts perform nonredundant, complementary services for their host. *B. cicadellinicola* synthesizes most of the sharpshooter's vitamins and cofactors, while *S. muelleri* appears to provide essential amino acids.

How three unrelated organisms manage to integrate such complex operations as metabolism and gene expression is a question for future study. But targeting any number of these essential bacterial pathways could prove a promising strategy for containing the spread of a potentially devastating pest—a risk perceived as so great that the sharpshooter is the only insect listed as a potential bioterrorism agent. And the researchers make a strong case that endosymbiont systems can help shed light on the molecular and evolutionary changes that allowed free-living organisms to take up residence in the cells of others—a path echoing the evolution of eukaryotes, whose cells contain mitochondria and chloroplasts, the descendants of ancient free-living bacteria.

## 

**Figure:**
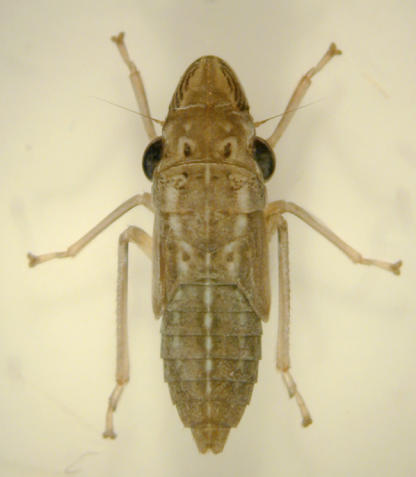
The genomes of two bacterial species that supply essential nutrients for the glassywinged sharpshooter provide potential targets for controlling infestations of this greatly feared agricultural pest

